# Positive and Negative Effects of Finance-based Social Capital on Incident Functional Disability and Mortality: An 8-year Prospective Study of Elderly Japanese

**DOI:** 10.2188/jea.JE20120025

**Published:** 2012-11-05

**Authors:** Naoki Kondo, Kohta Suzuki, Junko Minai, Zentaro Yamagata

**Affiliations:** 1 Department of Health Economics and Epidemiology Research, University of Tokyo School of Public Health, Tokyo, Japan; 2 東京大学大学院医学系研究科公共健康医学専攻臨床疫学・経済学分野; 2 Department of Health Sciences, Interdisciplinary Graduate School of Medicine and Engineering, University of Yamanashi, Chuo, Yamanashi, Japan; 1 山梨大学大学院医学工学総合研究部社会医学講座; 3 Department of Nursing, Odawara International University of Health and Welfare, Odawara, Kanagawa, Japan; 3 国際医療福祉大学小田原保健医療学部看護学科

**Keywords:** microfinance, ROSCA, *mujin*, social capital, activities of daily living

## Abstract

**Background:**

Rotating savings and credit associations (ROSCAs) involve group financial self-help activities. These voluntary financial cooperative associations—*mujin* in Japanese*—*are found in some rural areas of Japan. Cross-sectional evidence suggests that active participation in *mujin* correlates with rich social capital and better functional capacities among older adults. However, the effect of *mujin* on subsequent health outcomes is unknown.

**Methods:**

In 2003, we conducted a baseline interview survey of 583 functionally independent adults randomly selected from Yamanashi Prefecture residents aged 65 years or older. They were followed up until 2011. We used proportional hazards models, and factor analysis of 8 *mujin*-related questions identified 2 components: the “intensity and attitude” and “financing” aspects of *mujin*.

**Results:**

The hazard ratios (HRs) for incident functional disability—identified by using the public long-term care insurance database—per 1-SD increase in factor scores were 0.82 (95% CI: 0.68–0.99) for the intensity and attitude score and 1.21 (1.07–1.38) for financing score. Adjustments for age, sex, marital status, household composition, physical health, education, income, and other factor scores only slightly attenuated these HRs. The results for mortality models were very similar to those for incident functional disability.

**Conclusions:**

ROSCA-type activities in Japan could have beneficial effects on the health of older adults if used primarily for the purpose of friendship. *Mujin* for aggressively financial purposes might be somewhat harmful, as such activities might reflect the “dark side” of social capital, ie, overly demanding expectations of group conformity.

## Abstract (ja)

**【背景】:**

回転型貯蓄金融講（ROSCA）は金銭的な相互支援の活動である。日本では無尽（講）と呼ばれ、主に交流を目的としていくつかの地方で今も行われている。これまでに日本人高齢者の横断研究によって、無尽への活発な参加がソーシャル・キャピタルや機能維持と関連することが示されているが、健康との縦断的な関連についての報告はない。

**【方法】:**

山梨健康寿命追跡調査において、山梨県内に住む65歳以上の自立高齢者583名に訪問調査を実施した。2011年までの追跡調査結果を比例ハザードモデルを用いて分析した。無尽に関連する8個の質問を因子分析し、「（参加の）強度と態度」「金融的性質」の2因子を抽出した。

**【結果】:**

因子得点が1SD上昇するごとの、機能障害の発生（介護保険における要介護認定・要介護3以上の発生）のハザード比（HR）は「強度と態度」因子で0.82 (95%信頼区間: 0.68–0.99)、金融的性質因子で1.21 (1.07–1.38) であった。年齢、性別、婚姻状況、世帯構成、身体的健康状態、学歴、所得、もう一つの無尽因子で調整したところ、わずかにこれのハザード比は1に近づいた。死亡をアウトカムとし分析結果はほぼ同様であった。

**【結論】:**

日本のROSCAは、交流を目的としたものの場合高齢者の健康維持に良い影響をもたらす可能性がある。一方、金融目的の強い活動の場合は悪影響を与える可能性も示唆された。これは所属グループの規則への厳格な対応の必要性といったソーシャル・キャピタルの「負の側面」を反映している可能性がある。

## INTRODUCTION

Accumulating evidence suggests social capital is an important social determinant of individual health.^1^ According to a seminal work of Putnam,^2^ social capital refers to “features of social organization, such as trusts, norms, and networks, that can improve the efficiency of society by facilitating coordinated actions.” Putnam introduced the concept of the financial cooperative association, called a rotating savings and credit association (ROSCA), which is a form of informal local microfinance that builds strong structural social capital.^2^^,^^3^ In a ROSCA, members regularly make a fixed deposit at scheduled meetings, after which a different assigned member takes the aggregate deposit. This practice continues until each member has taken an aggregate deposit. Therefore, to be successful, ROSCA members must trust one another implicitly.^4^

Social capital should be developed within the intrinsic historical and cultural contexts of each community, to overcome shared challenges.^2^ As in many developing countries today, ROSCAs were very popular in Japan until the early postwar era. Japanese ROSCAs are called *mujin* in eastern Japan, *tanomoshi-ko* in western Japan, and *moai* in Okinawa Prefecture. Hereafter, for simplicity we will refer to all such organizations as *mujin* throughout this article.^5^^,^^6^ Although *mujin* have largely disappeared in Japan, they remain active in some rural and remote regions, including Yamanashi and Okinawa, where many communities are bound by strong cultural ties that allow them to share socioeconomic challenges.^7^ For example, in the Yamanashi area, 66% of older (age 65+ years) adults were currently or formerly engaged in *mujin*. The figure was 40% for the entire adult population. Among the older group, 70% have multiple *mujin* memberships, and 79% have continued to their *mujin* for longer than 10 years. Most (93%) *mujin* are held monthly; the mean deposit is 5703 Japanese yen and the mean party fee is 2311 yen. Payment and financing policies vary; in every round, a deposit taker is selected by auction or in a predetermined order. The taker may pay a fixed fee or a proportional amount of the bidding fee, which is used as interest for those who take deposits in a later round. However, this interest system has largely disappeared in many extant *mujin*.

Nevertheless, unlike traditional ROSCAs, many extant *mujin* focus less on the financial needs of their members than on the need to promote friendship and social bonding among members.^8^^,^^9^ For most (95%) participants, *mujin* are enjoyable, an important source of information, and essential to daily living and health.

In 2003, the results of a survey in the Yamanashi area showed a positive cross-sectional association between *mujin* participation and functional capacity among elderly Japanese (≥65 years).^9^ The same study proposed that, as a highly homogeneous group, *mujin* were a form of structural social capital that fostered strong membership bonds. However, this evidence was obtained from a cross-sectional analysis that was subject to reverse causation (ie, healthier individuals might be likelier to participate in *mujin*). Thus, the prospective association between *mujin* participation and subsequent health needs to be studied. In addition, because *mujin* activities can have various functional aspects, there is a need to evaluate whether different aspects of *mujin* have independent effects on the health of elderly Japanese. For example, Kawachi et al suggested the possibility of negative effects of social capital on health if group membership requires unfavorable conformity.^10^

In the present study, we used 8-year prospective data from the Yamanashi Healthy-Active Life Expectancy (Y-HALE) study to investigate the independent effects of multiple aspects of *mujin* on onset of functional disability and mortality in Yamanashi Prefecture, Japan. We studied elderly Japanese (age ≥65 years) because social capital is particularly important for that age group because of their close bonds with community members and because the probability of failing health increases with age.^10^ Because society is aging at an unprecedented pace in Japan and many other countries,^11^ postponement of long-term care (LTC) is a critical public health concern worldwide.

Our hypotheses were as follows: (1) because *mujin* strongly foster bonding among members, intensive participation in *mujin* is beneficial to the health of elders in terms of maintaining functional capacity and longevity independent of sociodemographic characteristics, (2) the beneficial health effects of *mujin* are explained by differences in the financial condition and health behavior of participants and nonparticipants, and (3) the health effects of *mujin* differ according to their components, ie, some components may have detrimental effects.

## METHODS

### Study participants

A full description of the sampling procedure for the Y-HALE study has been published elsewhere.^9^^,^^12^ In short, baseline data were collected from a 2-stage probability sample of 1800 older adults (≥65 years) who resided in Yamanashi Prefecture in 2002. In 2003, more-detailed questions were asked to 583 randomly selected participants who agreed to participate in this second survey. They were physically and cognitively independent in undertaking activities of daily living (ADL), ie, they were not qualified to receive public LTC insurance benefits. The municipal government determines applicant eligibility for LTC insurance benefits based on physical and cognitive functions, using a standardized procedure that includes a physician examination, computer-based provisional judgment, and a final decision made by a local insurance committee.^13^ Therefore, at baseline, we excluded residents who qualified for insurance benefits, as they already had ADL disabilities. Trained investigators visited the participants and gathered information on sociodemographic characteristics, social activities, diet, psychosocial factors, social capital, and health status. The response rate was 98%. After excluding participants with missing data for key measures, including age, sex, and number of household members, we analyzed data from 562 respondents (96%) to the 2003 survey. This study was approved by the Ethics Board of the School of Medicine, University of Yamanashi, and conforms to the principles of the Declaration of Helsinki.

### Follow-up

We conducted yearly follow-up consultations by mail or, when requested, by telephone interview. Participants reported health status, including recent qualifications for LTC insurance benefits (dates of qualification and levels of benefits for which they qualified). For participants who died, we asked their family members to report the date of death and cause of death. We confirmed responses concerning qualification for benefits by phoning participants’ officially assigned care-plan managers. To remain apprised of deaths, we also used alternate sources, such as death announcements in local newspapers, which covered 90% of deaths reported by participants’ family members. If we discovered or suspected that a participant had died, we mailed or phoned the person’s family to confirm the accuracy of the information. Participants who withdrew from the study because they did not want to participate, and those who could not be reached for any reason (such as departure from the study area), were treated as censored.

### Onset of functional disability

Japanese public LTC insurance has a 7-level authorization system that is based on the severity of physical and cognitive disabilities. This system is used to determine maximum usage of LTC services. In this study, we defined onset of functional disability as the point when a participant was certified as having a level 3 or greater LTC need. This level was selected because there is a clear boundary in disability severity between levels 2 and 3: people at level 2 or lower require only partial help to fulfill their basic ADL, such as toileting and bathing, whereas those at level 3 or greater are completely dependent on assistance for many ADL (Table 1). Also, using level 3 as a cutoff minimized the potential for bias from participants who did not apply for LTC care services because they already had sufficient informal support for their care needs. Participants with level 3 certification require full ADL support, and it is thus far less likely that they would lack a level 3 or higher certification, because once people start using LTC care services, they are likely to obtain the necessary certification levels that meet actual care needs so as to maximize the available monthly cost limit for services covered by LTC insurance.

**Table 1.** Definitions of levels used in the Japanese public long-term care insurance system

Level	ADL condition	Available services	Available monthly cost limit for service (Japanese yen)
Preventive-1/ Preventive-2	LTC is needed for some aspects of daily living, but proper care can improve or maintain ADL	LTC prevention programs	49 700/104 000

LTC-1	Unstable in rising and gait; partial support needed in toileting, bathing, etc.	Home-visit care, facility-based services	165 800

LTC-2	Difficulty in rising and gait; partial or complete support needed in toileting, bathing, etc.	Home-visit care up to 3 times a week, or facility-based services	194 800

LTC-3	Impossible to rise and no gait. Complete support needed in toileting, bathing, dressing, and all other basic ADL	Home-visit care during day and evening, home-visit intensive nursing care, or facility rehabilitation services (1 or 2 service times per day)	267 500

LTC-4	Severe decline in ADL capacity; complete support needed in toileting, bathing, dressing, and all other basic ADL	Home-visit care during day and evening, home-visit intensive nursing care, or facility rehabilitation services (2 or 3 service times per day)	306 000

LTC-5	Complete support needed in all ADL; difficulty with communication	Home-visit care during day and evening, home-visit intensive nursing care, or facility rehabilitation services (3 or 4 service times per day)	358 300

ADL: activities of daily living, LTC: long-term care.

### Scoring *mujin* domains

Participants were asked about their history of participation in *mujin*, number of current *mujin* memberships, current average frequency of participation in *mujin* meetings per month, maximum number of *mujin* memberships held at 1 time, and duration, frequency, type, financing deposit, and party fee for their primary *mujin* group. Complete information on the questionnaire regarding *mujin* has been published elsewhere.^9^ We conducted a factor analysis of these questions by using principal component methods. We used promax rotation to address correlations between factors and decided the number of factors on the basis of standard criteria, namely, by referring to the eigenvalue and evaluating the internal consistency of primary questions for each factor. To maximize data available for factor analysis, we imputed the mean value of each variable for missing observations. Although we gathered additional information on *mujin* characteristics, such as major topics of conversation in *mujin* and demographic characteristics of members, we did not use these data in our factor analysis because this additional information represented categorical variables that either could not be dealt with using a conventional factor analysis technique or did not compose a valid factor that could be reasonably characterized.

Rather than analyzing the indirect contextual social capital effects of *mujin* group involvement, we evaluated the direct effects of *mujin* on participant health because the contextual effect of *mujin* should be evaluated by using the *mujin* as the unit rather than by the geographical or administrative community unit, though we did not have any relevant information on the *mujin* groups in which the respondents participated. However, the importance of determining the effect of individual social capital rather than that of aggregated social capital is evident, as social capital measured at the individual level collectively constitutes social capital at the community level.^14^

### Covariates

As seen in Table 2, the covariates used included demographic variables (age, sex, marital status, household size), physical health (Physical Component Summary [PCS] of the Medical Outcomes Study Short Form-36 [SF-36], Japanese version 1.2),^15^ socioeconomic status (educational attainment and individual income), levels of social activity, and health behaviors (smoking habit, alcohol consumption, and exercise habits). Social activity was measured using the question, “How frequently do you participate in the following community activities (community festivals, residents’ associations, senior citizens’ clubs, hobby activities, volunteer activities, and activities based on special/traditional skills)?”. This Japanese social activity scale for older people was validated by Ohno^16^ and allowed us to determine whether a participant was socially inactive, normal, active, or very active.

**Table 2.** Incidence of functional disability and mortality by characteristics of adults aged 65 years or older in Yamanashi, Japan (2003)

Variable (Number of missing data)	Baseline		Functional disability			Mortality		
		
	*n* (%) or mean [SD]		No.	Person- years	Incidence rate	No.	Person- years	Incidence rate
Sex (0)								
Female	267	(48)	46	1806.6	0.025	27	2002.5	0.013
Male	295	(52)	80	1913.6	0.042	68	2051.2	0.033
Age, y (0)								
65–74	250	(44)	21	1772.9	0.012	19	1861.3	0.010
75+	312	(56)	105	1947.3	0.054	76	2192.4	0.035
Marital status (0)								
Married	397	(71)	79	2648.8	0.030	65	2869.6	0.023
Not married	165	(29)	47	1071.4	0.044	30	1184.1	0.025
Household size (0)								
1	51	(9)	7	332.4	0.021	5	375.9	0.013
2	208	(37)	40	1395.2	0.029	33	1508.7	0.022
3+	303	(54)	79	1992.6	0.040	57	2169.2	0.026
Average number of household members (0)	3.2	[1.8]	—	—	—	—	—	—
SF-36 (PCS) score (3)	51.1	[8.9]	—	—	—	—	—	—
Educational attainment (66)								
Less than high school	210	(42)	53	1351.2	0.039	39	1501.3	0.026
High school or higher	286	(58)	57	1954.6	0.029	42	2085.2	0.020
Annual individual income^a^ (80) (Japanese yen)			—	—	—	—	—	—
Women	46.9	[14.5]						
Men	136.5	[8.7]	—	—	—	—	—	—
Smoking (1)								
Never	328	(58)	69	2183.2	0.032	47	2403.0	0.020
Ex-smoker	163	(29)	40	1081.3	0.037	33	1151.2	0.029
Current smoker	70	(12)	17	451.4	0.038	15	491.9	0.030
Alcohol consumption (g/day) (0)								
None	283	(50)	72	1848.9	0.039	51	2042.6	0.025
0–20	66	(12)	22	414.2	0.053	19	445.8	0.043
21–40	121	(22)	21	806.8	0.026	18	870.7	0.021
41+	92	(16)	11	650.2	0.017	7	694.6	0.010
Exercise habit (1)								
Yes	427	(76)	90	2867.1	0.031	69	3106.7	0.022
No	134	(24)	36	845.3	0.043	26	939.3	0.028

^a^Geometric mean.

### Statistical analysis

Using the statistical analysis software package SAS version 9.2, we conducted Cox proportional hazards regression and modeled the principal component scores for *mujin* with covariates. We visually and statistically confirmed a proportional hazard assumption. We created 2 separate models, with onset of functional disability (or being certified for level 3 or greater LTC need) and mortality as outcomes. In the disability models, all deceased cases were treated as censored.

## RESULTS

In total, we observed 4055 person-years. During 8 years of follow-up, 126 participants developed a level 3 or greater LTC need and another 95 died, including 11 who had received level 3 or greater LTC certification before death. There were 56 dropouts (ie, individuals who refused to participate in further studies and became nonresponsive). The incidence rates of onset of functional disability and mortality varied with respect to participant socioeconomic status and social capital (Tables 2 and 3).

**Table 3.** Incidence of functional disability and mortality by baseline *mujin* characteristics among 562 adults aged 65 years or older in Yamanashi, Japan (2003)

	*n* (%) or mean [SD]		Functional disability			Mortality		
		
			No.	Person- years	Incidence rate	No.	Person- years	Incidence rate
Participating in *mujin*								
0. Never	235	(42)	56	1521.5	0.037	42	1681.3	0.025
1. Past	137	(24)	38	897.1	0.042	28	973.9	0.029
2. Current	190	(34)	32	1301.6	0.025	25	1398.5	0.018
Maximum number of concurrent *mujin* memberships	2.3	[1.6]	—	—	—	—	—	—
Duration, y^a^	19.6	[13.3]	—	—	—	—	—	—
Frequency of meeting^a^								
1. Bimonthly or less	22		5	169.2	0.0296	4	177.3	0.0226
2. Monthly or more	303		65	2029.5	0.032	49	2195.1	0.0223
Is *mujin* fun or not^a^								
1. Not so much fun/not fun at all	16		6	108.7	0.055	4	114.5	0.035
2. Somewhat fun	89		20	595.0	0.034	14	637.9	0.022
3. Great fun	218		44	1466.4	0.030	35	1589.0	0.022
Size (number of members)^a^	6.8	[7.2]	—	—	—	—	—	—
Cost per meeting for meals and drinks (party fee) per time (Japanese yen)^a^	2311	[1808]	—	—	—	—	—	—
Deposit for group financing per time (Japanese yen)^a^	5703	[7718]	—	—	—	—	—	—

^a^For the *mujin* meeting that is most important for the respondent (if he/she participates in >1 *mujin* group).

The variables used for factor analysis were correlated with each, and correlation coefficients ranged between 0.49 and 0.94 (Table 4), excepting deposit for group financing, which was more weakly correlated with the other variables (correlation coefficient, 0.33–0.43). Factor analysis identified 1 component from the 8 questions on *mujin* (Table 5). We called this “intensity and attitude” toward *mujin*, which was reflected in all questions except those querying the amount of the deposit for group financing.

**Table 4.** Correlation coefficients for *mujin* variables

Variable	Variable number							

	1	2	3	4	5	6	7	8
1. Participation in *mujin*	1	0.70	0.75	0.88	0.90	0.72	0.63	0.42
2. Maximum number of concurrent *mujin* memberships		1	0.62	0.66	0.66	0.59	0.52	0.43
3. Duration (y)			1	0.66	0.70	0.61	0.49	0.43
4. Meeting frequency				1	0.94	0.77	0.62	0.42
5. Is *mujin* fun or not?					1	0.76	0.65	0.44
6. Size (number of members)						1	0.52	0.33
7. Cost per meeting for meals and drinks (party fee)							1	0.36
8. Deposit for group financing								1

All coefficients are statistically significant (*P* < 0.001).

**Table 5.** Standardized scoring coefficients yielded by factor analysis of *mujin* characteristics: principal component method with promax rotation

Variable	Factor 1 (intensity and attitude)	Factor 2 (financing)
Participation in *mujin*	0.200	−0.026
Maximum number of concurrent *mujin* memberships	0.140	0.164
Duration (y)^a^	0.151	0.127
Meeting frequency^a^	0.200	−0.047
Is *mujin* fun or not?^a^	0.179	−0.053
Size (number of members)^a^	0.192	−0.134
Cost per meeting for meals and drinks (party fee) for each member^a^	0.149	0.026
Deposit for group financing^a^	−0.037	0.946

Eigenvalue	5.144	0.762
Cronbach’s alpha (for variables with |score| > 0.1)	0.926	0.801
Cumulative contribution = 73.8%		

^a^For the *mujin* meeting that is most important for the respondent (if he/she participates in >1 *mujin* group).

Moreover, although the eigenvalue was not large (0.762), we adopted another factor to which the amount of the deposit for group financing made a significant contribution. We accepted this second factor because it may reflect the financing aspect of *mujin*, which can be distinguished from intensity and attitude. We refer to this factor as “financing.”

The results of proportional hazards regression showed that the hazard ratio (HR) per 1-SD increase in the *mujin* component for intensity and attitude was 0.82 (95% CI: 0.68–0.99; Table 6). In contrast, the HR for the “financing” aspect of the *mujin* score was higher than unity (1.21, 95% CI: 1.07–1.38). These HRs were slightly altered by adjustment for demographics, physical health, educational attainment, and income (Model 1 in Table 6). In addition, when adjusting for social activity score, the adjusted HR for intensity and attitude was largely attenuated, to 1.01 (0.81–1.25), whereas that for the financing aspect remained statistically significant, at 1.20 (1.07–1.35; Model 2). As indicated by the results of Model 3, further adjustments for health behavior did not alter the HRs for either *mujin* factor. The results of bivariate proportional hazards models for mortality were almost identical to those for the onset of functional disability (Table 7). Although HRs were not statistically significant in multivariate models, the financing aspect was similarly associated with mortality, with adjusted HRs ranging between 1.09 and 1.11. In contrast, the adjusted HRs for intensity and attitude were very close to unity.

**Table 6.** Hazard ratios (95% CI) for onset of functional disability

	Bivariate	Model 1	Model 2	Model 3
*Mujin*				
Intensity and attitude^a^	0.82 (0.68–0.99)	0.88 (0.72–1.08)	1.01 (0.81–1.25)	1.03 (0.82–1.28)
Financing^a^	1.21 (1.07–1.38)	1.18 (1.06–1.31)	1.20 (1.07–1.35)	1.18 (1.05–1.33)
Age: 75+ (vs <75)	4.69 (2.93–7.49)	4.26 (2.52–7.22)	4.78 (2.75–8.33)	5.06 (2.88–8.87)
Male (vs female)	1.66 (1.16–2.39)	1.80 (1.16–2.77)	1.60 (0.99–2.59)	2.56 (1.34–4.90)
Having spouse: no (vs yes)	1.47 (1.02–2.11)	1.64 (0.98–2.75)	1.75 (1.01–3.04)	2.00 (1.12–3.57)
Household members (base: 3+ people)				
Living alone	0.53 (0.24–1.15)	0.50 (0.17–1.47)	0.49 (0.20–1.20)	0.57 (0.23–1.42)
2	0.72 (0.49–1.06)	0.96 (0.57–1.60)	1.03 (0.61–1.74)	1.14 (0.67–1.95)
Physical health (SF-36, PCS score)^a^	0.80 (0.68–0.94)	0.82 (0.68–0.99)	0.83 (0.69–1.01)	0.87 (0.71–1.06)
Education: high school graduate or higher (vs less than high school graduate)	0.74 (0.51–1.07)	0.92 (0.57–1.50)	0.92 (0.61–1.39)	0.98 (0.64–1.50)
Income (log-transformed: yen/month)	0.99 (0.92–1.07)	1.03 (0.93–1.15)	1.04 (0.93–1.17)	1.06 (0.94–1.19)
Social activity (base: not active)				
Normal	0.43 (0.27–0.67)		0.55 (0.32–0.93)	0.54 (0.31–0.92)
Active	0.32 (0.20–0.53)		0.32 (0.18–0.56)	0.30 (0.17–0.54)
Very active	0.36 (0.19–0.70)		0.34 (0.16–0.73)	0.33 (0.15–0.72)
Smoking habits (Base: Never)				
Ex-smoker	1.18 (0.80–1.74)			0.68 (0.37–1.25)
Current smoker	1.20 (0.70–2.04)			1.11 (0.53–2.36)
Alcohol consumption (Base: nondrinker; g/day)				
1–20	1.38 (0.86–2.23)			1.12 (0.61–2.05)
21–40	0.67 (0.41–1.08)			0.55 (0.30–1.01)
40+	0.43 (0.23–0.81)			0.39 (0.18–0.84)
Exercise habits (yes) (vs no)	1.04 (0.99–1.08)			0.96 (0.90–1.01)

^a^HR is per 1-SD increase.

**Table 7.** Hazard ratios (95% CI) for all-cause mortality

	Bivariate	Model 1	Model 2	Model 3
*Mujin*				
Intensity and attitude^a^	0.87 (0.70–1.08)	1.00 (0.78–1.29)	1.04 (0.80–1.34)	1.08 (0.83–1.39)
Financing^a^	1.16 (1.05–1.29)	1.09 (0.99–1.21)	1.11 (0.99–1.24)	1.09 (0.97–1.22)
Age: 75+ (vs <75)	3.45 (2.09–5.71)	2.92 (1.64–5.22)	3.13 (1.73–5.66)	3.41 (1.86–6.24)
Male (vs female)	2.51 (1.60–3.91)	2.04 (1.16–3.58)	2.18 (1.21–3.92)	3.18 (1.48–6.81)
Having spouse: no (vs yes)	1.12 (0.73–1.73)	1.49 (0.78–2.86)	1.58 (0.82–3.07)	1.82 (0.91–3.65)
Household members (base: 3+ people)				
Living alone	0.50 (0.20–1.26)	0.50 (0.17–1.47)	0.53 (0.18–1.57)	0.65 (0.21–1.98)
2	0.83 (0.54–1.27)	0.96 (0.57–1.60)	1.10 (0.62–1.96)	1.20 (0.66–2.17)
Physical health (SF-36, PCS score)^a^	0.83 (0.69–1.01)	0.84 (0.67–1.06)	0.85 (0.67–1.06)	0.88 (0.70–1.11)
Education: high school graduate or higher (vs less than high school graduate)	0.77 (0.50–1.20)	0.92 (0.57–1.50)	0.97 (0.60–1.57)	1.11 (0.67–1.83)
Income (log-transformed: yen/month)	1.01 (0.92–1.11)	1.01 (0.90–1.14)	1.02 (0.90–1.15)	1.03 (0.91–1.16)
Social activity (base: not active)				
Normal	0.52 (0.31–0.88)		0.61 (0.32–1.17)	0.64 (0.33–1.24)
Active	0.42 (0.23–0.75)		0.43 (0.22–0.84)	0.42 (0.21–0.84)
Very active	0.45 (0.21–0.95)		0.40 (0.16–0.99)	0.42 (0.16–1.05)
Smoking habits (Base: Never)				
Ex-smoker	1.48 (0.95–2.32)			0.79 (0.39–1.59)
Current smoker	1.58 (0.88–2.82)			1.27 (0.55–2.93)
Alcohol consumption (Base: nondrinker; g/day)				
1–20	1.73 (1.02–2.94)			1.41 (0.72–2.75)
21–40	0.83 (0.48–1.41)			0.57 (0.28–1.14)
40+	0.40 (0.18–0.88)			0.31 (0.12–0.79)
Exercise habits (yes) (vs no)	1.03 (0.98–1.08)			0.97 (0.90–1.04)

^a^HR is per 1-SD increase.

## DISCUSSION

In a representative sample of elderly residents of Yamanashi, we found that individuals who intensively and enthusiastically participated in *mujin* were more likely to maintain functional capacity. The protective effect of *mujin* activities can be partially explained by socioeconomic status and fully explained by social activity in general. However, the contribution of health behavior to explaining the effect of *mujin* was not as strong as we expected. A striking finding of our study was that participation in *mujin* that were actively involved in group financing was positively associated with incident disability, suggesting that such *mujin* might be hazardous to health.

In recent years, microfinance—one of the more famous examples being the Grameen Bank in Bangladesh^17^—has emerged as a financial intervention in impoverished rural communities in developing countries. Some, but not all, research indicates that microfinance has greatly contributed to eliminating poverty and improving health and health behavior by empowering group members and strengthening social capital.^18^^–^^20^ Although, strictly speaking, microfinance managed by commercial banks or nongovernmental organizations is not a term formally applied to structures such as ROSCAs, the two nevertheless share many characteristics in terms of the roles of individual members within the group. Thus, the findings of our study are consistent with those of studies on microfinance.

Individual social capital could be the vector of various forms of social support.^12^ Our prospective evidence suggests that *mujin* activities serve the community by fostering strong individual (structural) social capital^2^ and protecting elderly adults against progression of what would otherwise be the natural course of ADL deterioration. In the present study, adjustment for the level of social activity support attenuated the beneficial effect of *mujin*, which suggests that *mujin* have a role similar to social activity in general, which is a known component of structural individual social capital. Health behavior did not fully explain the association between enthusiastic intensive participation in *mujin* and lower incidence of disability, which suggests that psychosocial support (such as the provision of emotional support, prevention of social isolation, and maintenance of morale) is the central role of *mujin* rather than promoting healthier behavior (such as smoking cessation and regular exercise), at least for older Japanese adults.^21^

It is especially interesting that we also found a higher health risk associated with the financial aspect of *mujin*. This is probably a reflection of the “dark side” of social capital.^10^ According to Kawachi et al, this potential negative aspect of social capital includes

(a) excessive demands placed upon members of cohesive groups to provide support to others, (b) expectations of conformity that may result in restrictions on individual freedom as well as intolerance of diversity, (c) the exercise of in-group solidarity to exclude members of out-groups, and (d) down-leveling of norms within a tightly knit group, which may obstruct potential upward social mobility” (Kawachi et al, 2008; page 5).^10^

Given the important responsibility associated with financial transactions among *mujin* members, it is logical to presume that members are subject to high demands for and expectations of conformity to the wishes of other group members.^5^ In fact, ethnological researchers have described the strongly closed nature of “serious” *mujin* and have suggested potential negative effects on group members due to the daunting scale of mutual responsibility.^22^^,^^23^

The validity and reliability of this study are supported by the very high response rate, the use of highly standardized outcome measures, and the factor analysis-based scoring approach used to evaluate social capital and *mujin*. Nevertheless, some limitations of this study should be noted. First, our definition of disability onset may differ from actual levels of disability. However, we addressed this in part by specifying the need for level 3 LTC as the cutoff for having full ADL support. Similarly, although we used multiple approaches to ensure the accuracy of information on participant deaths, our strategy to identify deceased cases did not rely on official death records, potentially causing information biases. For example, some decedents might have been treated as censored, which would result in underestimation of the number of deaths and lower statistical power in our analyses. Second, the evaluation of 2 *mujin* factors might not be sufficiently valid because the variables in our factor analysis included both continuous and ordered specifications. However, their validity and reliability were partially supported by high internal consistency and the fact that our findings did not contradict those of previous theoretical and empirical studies.^2^^,^^9^ Finally, although some studies found sex differences in the association between social capital and health, our analyses were not stratified by sex due to the limited sample size.

In conclusion, because social capital is developed in specifically local cultural and historical contexts, determining the health effects of community-specific social capital—such as that afforded by *mujin*—would increase understanding for public health research on social capital. Our study of the Japanese equivalent of ROSCA activities among elderly adults shows that such financial self-help groups, which are common in many parts of the world, could have beneficial effects on member health. These effects may be due not only to financial empowerment, but also to strengthening of interpersonal ties. Nevertheless, our striking findings regarding the potential negative effect of *mujin* that have an aggressive financing role warrant further study. Understanding the potential positive and negative effects of social capital is critical, especially when promoting community-based financial interventions such as microfinance programs, which have been steadily gaining popularity worldwide.^18^^,^^19^

## ONLINE ONLY MATERIALS

The Japanese-language abstract for articles can be accessed by clicking on the tab labeled Supplementary materials at the journal website http://dx.doi.org/10.2188/jea.JE20120025.

Abstract in Japanese.
