# Supplementary material for: Positive and Negative Effects of Finance-based Social Capital on Incident Functional Disability and Mortality: An 8-year Prospective Study of Elderly Japanese
Source: J Epidemiol. 2012 Nov 5;22(6):543–50. doi: 10.2188/jea.JE20120025 (PMC3798567; doi:10.2188/jea.JE20120025)
Supplement: Abstract in Japanese. [file je-22-543-s001.pdf]

経済的な相互支援を目的としたソーシャル・キャピタル（無尽講）が機能障害及び死亡のリスクに与える影響：8 年間の追跡研究

近藤尚己[1,2]，鈴木孝太[1]，薬袋淳子[3]，山縣然太朗[1]

[1] 山梨大学大学院医学工学総合研究部社会医学講座

[2] 東京大学大学院医学系研究科公共健康医学専攻臨床疫学・経済学分野

[3] 国際医療福祉大学小田原保健医療学部看護学科

【背景】回転型貯蓄金融講（ROSCA）は金銭的な相互支援の活動である。日本では無尽（講）と呼ばれ、主に交流を目的としていくつかの地方で今も行われている。これまでに日本人高齢者の横断研究によって、無尽への活発な参加がソーシャル・キャピタルや機能維持と関連することが示されているが、健康との縦断的な関連についての報告はない。

【方法】山梨健康寿命追跡調査において、山梨県内に住む 65 歳以上の自立高齢者 583 名に訪問調査を実施した。2011 年までの追跡調査結果を比例ハザードモデルを用いて分析した。無尽に関連する 8 個の質問を因子分析し、「(参加の) 強度と態度」「金融的性質」の 2 因子を抽出した。

【結果】因子得点が 1 SD 上昇するごとの、機能障害の発生（介護保険における要介護認定・要介護 3 以上の発生）のハザード比（HR）は「強度と態度」因子で 0.82（95%信頼区間: 0.68-0.99）、金融的性質因子で 1.21（1.07-1.38）であった。年齢、性別、婚姻状況、世帯構成、身体的健康状態、学歴、所得、もう一つの無尽因子で調整したところ､わずかにこれのハザード比は 1 に近づいた。死亡をアウトカムとし分析結果はほぼ同様であった。

【結論】 日本の ROSCA は、交流を目的としたものの場合高齢者の健康維持に良い影響をもたらす可能性がある。一方、金融目的の強い活動の場合は悪影響を与える可能性も示唆された。これは所属グループの規則への厳格な対応の必要性といったソーシャル・キャピタルの「負の側面」を反映している可能性がある。
